# Quality of Physical Activity Apps: Systematic Search in App Stores and Content Analysis

**DOI:** 10.2196/22587

**Published:** 2021-06-09

**Authors:** Sarah Paganini, Yannik Terhorst, Lasse Bosse Sander, Selma Catic, Sümeyye Balci, Ann-Marie Küchler, Dana Schultchen, Katrin Plaumann, Sarah Sturmbauer, Lena Violetta Krämer, Jiaxi Lin, Ramona Wurst, Rüdiger Pryss, Harald Baumeister, Eva-Maria Messner

**Affiliations:** 1 Department of Sport Psychology Institute of Sports and Sport Science Albert-Ludwigs-University Freiburg Freiburg Germany; 2 Department of Clinical Psychology and Psychotherapy Institute of Psychology and Education Ulm University Ulm Germany; 3 Department of Rehabilitation Psychology and Psychotherapy Institute of Psychology Albert-Ludwigs-University Freiburg Freiburg Germany; 4 Department of Clinical and Health Psychology Institute of Psychology and Education Ulm University Ulm Germany; 5 Institute of Media Informatics Ulm University Ulm Germany; 6 Chair of Health Psychology Department of Psychology Friedrich-Alexander Universität Erlangen-Nürnberg Erlangen Germany; 7 Department of Psychiatry and Psychotherapy Medical Center Faculty of Medicine University of Freiburg Freiburg Germany; 8 Institute of Clinical Epidemiology and Biometry University of Würzburg Würzburg Germany

**Keywords:** sports, exercise, mobile apps, mHealth, quality indicators, systematic review

## Abstract

**Background:**

Physical inactivity is a major contributor to the development and persistence of chronic diseases. Mobile health apps that foster physical activity have the potential to assist in behavior change. However, the quality of the mobile health apps available in app stores is hard to assess for making informed decisions by end users and health care providers.

**Objective:**

This study aimed at systematically reviewing and analyzing the content and quality of physical activity apps available in the 2 major app stores (Google Play and App Store) by using the German version of the Mobile App Rating Scale (MARS-G). Moreover, the privacy and security measures were assessed.

**Methods:**

A web crawler was used to systematically search for apps promoting physical activity in the Google Play store and App Store. Two independent raters used the MARS-G to assess app quality. Further, app characteristics, content and functions, and privacy and security measures were assessed. The correlation between user star ratings and MARS was calculated. Exploratory regression analysis was conducted to determine relevant predictors for the overall quality of physical activity apps.

**Results:**

Of the 2231 identified apps, 312 met the inclusion criteria. The results indicated that the overall quality was moderate (mean 3.60 [SD 0.59], range 1-4.75). The scores of the subscales, that is, *information* (mean 3.24 [SD 0.56], range 1.17-4.4), *engagement* (mean 3.19 [SD 0.82], range 1.2-5), a*esthetics* (mean 3.65 [SD 0.79], range 1-5), and *functionality* (mean 4.35 [SD 0.58], range 1.88-5) were obtained. An efficacy study could not be identified for any of the included apps. The features of data security and privacy were mainly not applied. Average user ratings showed significant small correlations with the MARS ratings (*r*=0.22, 95% CI 0.08-0.35; *P*<.001). The amount of content and number of functions were predictive of the overall quality of these physical activity apps, whereas app store and price were not.

**Conclusions:**

Apps for physical activity showed a broad range of quality ratings, with moderate overall quality ratings. Given the present privacy, security, and evidence concerns inherent to most rated apps, their medical use is questionable. There is a need for open-source databases of expert quality ratings to foster informed health care decisions by users and health care providers.

## Introduction

Physical inactivity is a significant risk factor for noncommunicable diseases such as cancer, diabetes, cardiovascular diseases, or chronic respiratory diseases and is estimated to cause 6%-10% of these diseases worldwide [1,2]. Insufficient physical activity is also a leading risk factor for mortality and was reported to be associated with 9% of premature death cases in 2008 [2]. The World Health Organization recommends at least 150 minutes of moderate or 75 minutes of vigorous-intensity physical activity per week for adults [3]. However, about 30% of adults do not follow this recommendation and are physically inactive [4].

Evidence indicates that regular physical activity results in physical, social, and mental health benefits such as better quality of sleep, lower depressive symptomatology, higher well-being, and a reduced risk of a large number of noncommunicable diseases [5-7]. Mobile apps might be a cost-effective and scalable option to foster behavior change in daily life [8]. Apps can also be beneficial as a supplement to behavioral interventions [9]. Additionally, fitness apps are very popular in the general population; a survey conducted in the United States in 2015 showed that about 58% of mobile phone users had downloaded a health app [10]. Of these, the most common categories were fitness and nutrition apps, and most respondents were using them daily. Moreover, health app users were more likely to meet the World Health Organization recommendations concerning physical activity [3,11] and apps were found to be efficacious in promoting physical activity with moderate effect sizes [12]. In 2 recent meta-analyses of apps for increasing physical activity, there was an increase in objectively measured physical activity in the app groups compared to that in the control groups [13,14]. However, these differences were not significant. Regarding the content and quality of apps promoting physical activity, previous reviews focused mainly on the use of behavioral change techniques (BCTs) developed by Abraham and Michie [15-21]. The most often provided BCTs were feedback on performance, self-monitoring, and goal setting [15,16,18,19].

Regarding the quality of apps, Schoeppe and colleagues [22] used the standardized Mobile App Rating Scale (MARS) [23] to evaluate apps for improving diet, physical activity, and sedentary behavior. However, the mentioned reviews show some limitations as these reviews mostly evaluated apps with specific characteristics (eg, only apps that are connected to an electronic activity monitor) [18], apps especially developed for children and adolescents [20,22], a limited number of apps (eg, only the 20 top-ranked apps, random selection of apps, or apps with a star-rating of at least 4) [16,19,22], or apps with certain contents and features (eg, only apps with feedback and apps that follow the official World Health Organization recommendation for physical activity) [15]. Overall, most studies evaluating apps for health behavior change use self-developed evaluation checklists and do not assess privacy and security features [24]. The only validated evaluation tools that were used are the BCT taxonomy and, in a few studies, the MARS [15,16,18,22,24].

Besides the evaluation of theory-based content, applied techniques or functions, and effectiveness, it is important to consider the risks of mobile health app use, such as inadequate protection of data and privacy or lack of informed consent [24,25]. Many physical activity apps are available, particularly via the 2 largest app stores, Google Play store and the App Store but there is only limited information on the quality and data security of these apps [24,26]. Initial studies concerning data security of medicine-related, depression, and smoking cessation apps reveal worrying results as sharing medical health data with third parties is routine and mostly not made transparent [27,28]. The only study evaluating the safety of personal data in physical activity apps also revealed substantial shortcomings [19]. In terms of quality, user ratings seem to be a questionable indicator as they seem to be mostly influenced by usability and functionality [26,29]. However, a recent evaluation revealed a positive correlation between a broad range of app quality ratings and user star ratings [30].

The aim of this study was to conduct a systematic and objective investigation of the physical activity apps available in 2 major app stores by using the German version of MARS (MARS-G). The MARS-G is a multidimensional instrument specifically developed to assess app quality on the dimensions engagement, functionality, aesthetics, and information quality [31]. Furthermore, privacy and security measures as well as the general characteristics and functions of physical activity apps will be assessed. The following research questions are addressed:

1. What is the quality of the apps promoting physical activity regarding engagement, functionality, aesthetics, and information?
2. What are the general characteristics, content, functions, privacy, and security measures of the apps promoting physical activity?
3. Are the user ratings in agreement with the expert quality ratings?
4. Which app features can predict app quality?

## Methods

### Search Strategy and Eligibility Criteria

A web crawler (automated web search engine) was used to scan the European Google Play store and App Store to search for eligible apps. The search was carried out on February 20, 2018 by using the following search terms: (1) active, (2) endurance, (3) exercise, (4) fitness, (5) gymnastics, (6) muscle, (7) shape, (8) strength, (9) training, and (10) workout. The search string to identify apps targeting physical activity was developed in an expert discussion (EMM and HB). The web crawler searches for each term and app store. Duplicates were automatically removed. After identification, a two-step procedure was applied by 2 independent researchers: (1) checking eligibility based on app title and description and (2) checking eligibility based on information in the downloaded app. In the first step, all identified apps were screened for whether their title, description, and images indicated that the app was developed for promoting physical activity (with at least 50% of the content focusing on physical activity); the app was available in German or English; the app was downloadable through the official Google Play store or App Store; the app could be used without further equipment, devices, or programs; and the app was primarily developed for adults. In the second step, all downloaded apps were assessed in detail to check whether they met the abovementioned eligibility criteria. If apps did not work after the download (checked with 2 different mobile phones) or were explicitly developed for children (explicitly stated in the title, description, or aims of the app), they were excluded. The other exclusion criteria were (1) app bundle, (2) only working with additional device (eg, Garmin connect), or (3) targeting specific person groups (eg, employees of a specific company).

### Rating Procedure

Each app was rated by 2 independent raters between February and October 2018. All raters undertook a free online training [32] (training module last updated on November 25, 2019). Raters were recruited from an interdisciplinary expert team (sports science, sport psychology, clinical psychology, information technology: EMM, YT, SP, LS, JL, SC, SB, LK, AK, DS, SS, KP, RP, and RW). Each app was tested and used for at least 15-20 minutes before the rating. The interrater reliability between the raters was computed for quality assurance.

### Outcome Measures

The MARS includes a multidimensional quality rating consisting of 4 dimensions: engagement (5 items: fun, interest, individual adaptability, interactivity, and target group), functionality (4 items: performance, usability, navigation, and gestural design), aesthetics (3 items: layout, graphics, and visual appeal), and information quality (7 items: accuracy of app description, goals, quality of information, quantity of information, quality of visual information, credibility, and evidence base) [23,31]. Hereby, the evidence base (dimension: information quality) was identified by app description and developer’s or provider’s websites. Items are rated from 1 (inadequate) to 5 (excellent). Besides these objective scales, subjective quality (recommendation, frequency of use, willingness to pay, overall star rating) and perceived impact on the user (awareness, knowledge, attitudes, intention to change, help-seeking, behavioral change) were assessed. Furthermore, the assessment includes a classification section to examine the app characteristics. The following variables were extracted: (1) app name, (2) store link, (3) platform (Google Play store and App Store), (4) content-related subcategory, (5) aims, (6) price, (7) user rating, (8) content, strategies, and functions (abbreviated as *functions* in the following, assessed with 22 items; ie, information/education, monitoring/tracking, goal setting, gamification, reminder) and (9) privacy and security features [23,31]. Privacy and security features were rated on a descriptive level (ie, presence of privacy policy, contact information or imprint, log-in with a password). Only information that was displayed within the app was used for evaluation. In this study, MARS-G was used [23,31]. The validation of the MARS-G yielded excellent internal consistency (ω=0.84, 95% CI 0.77-0.88) and high levels of interrater reliability (intraclass correlation coefficient [ICC] 0.83, 95% CI 0.82-0.85) [31].

### Statistical Analysis

To evaluate consistency, the ICC between the raters was calculated for quality assurance. Rater agreement was examined by ICC based on a two-way mixed-effects model [33]. An ICC of <0.50 is considered poor, 0.51-0.75 as moderate, 0.76-0.89 as good, and >0.90 as excellent [34]. A minimum ICC of 0.8 was predefined as a sufficient ICC in this study. For quality evaluation, means and standard deviations were calculated for each dimension of the MARS separately and overall. For all calculations, the mean of both raters was used. Further correlation between user ratings provided by Google Play/App Store and the MARS rating was calculated. For correlations analysis, an alpha level of 5% was defined. *P* values were adjusted using the procedure proposed by Holm [35]. To determine relevant predictors for overall quality, exploratory multiple linear regression analysis was conducted. Price, store, and the number of functions were used as predictors, as they were significant predictors in other systematic app reviews (eg, older adults, mindfulness, depression, posttraumatic stress disorder, rheumatoid arthritis) [36-40]. Dichotomous predictors were dummy coded. Regression estimates represent unstandardized regression coefficients.

## Results

### Search Results

**Figure 1:**
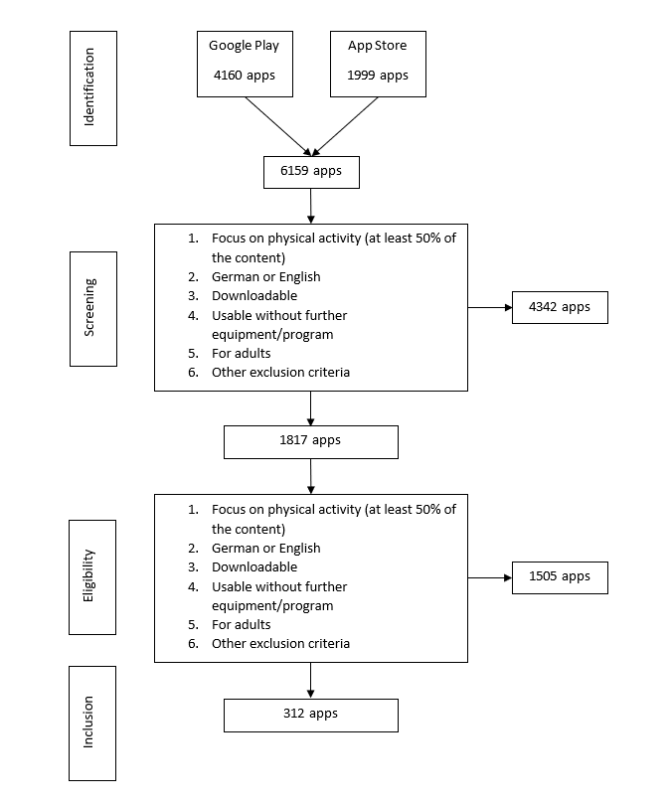
Flowchart of the inclusion and exclusion processes.

The search in the Google Play store and App Store yielded 6159 apps without duplicates. Screening resulted in the inclusion of 1817 apps. After downloading and assessing the eligibility criteria in detail, 1495 apps had to be excluded. The remaining 312 apps were included in the analyses (see Figure 1 for further information).

### App Characteristics

**Figure 2:**
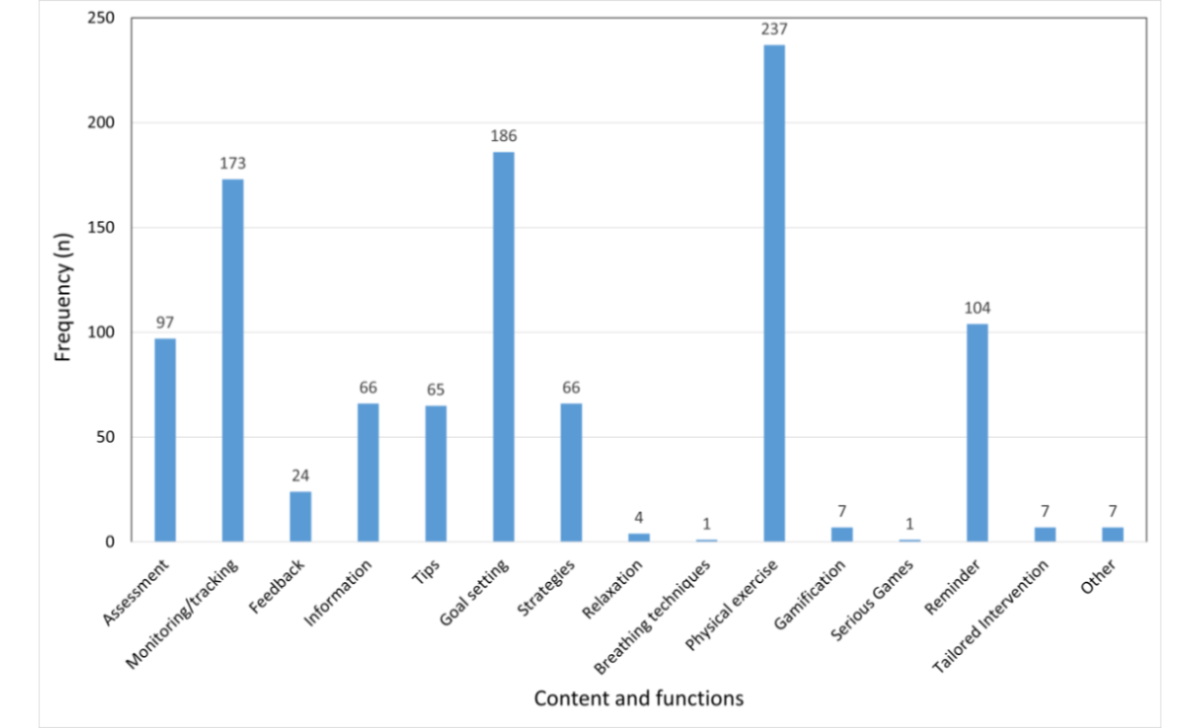
Content and functions of the included apps.

Of the 312 included apps, 143 (45.8%) were available in Google Play store and 169 (54.2%) were available in the App Store. The average user’s star rating was 4.29 (SD 0.70) (range 0-5). The costs for the included apps ranged from free for 96.8% (302/312) of the apps to €10.99 (mean cost €3.54 [SD 2.97]; €1=US $1.20). Regarding the store categories, the majority of the apps (300/312) were listed under health and fitness (multiple categories were assigned in stores). The further assigned categories were sports (n=83), lifestyle (n=69), business (n=4), entertainment (n=3), medicine (n=2), social (n=2), food and drink (n=1), and travel (n=1). Apps provided a broad range of functions (Figure 2). Most apps offered physical exercises (237/312, 75.9%) followed by goal setting (186/312, 59.6%) and monitoring/tracking (173/312, 55.4%). Apps further provided reminders (104/312, 33.3%), assessments (97/312, 31.1%), information (66/312, 21.2%), strategies/skills (66/312, 21.2%), tips/advice (65/312, 20.8%), feedback (24/312, 7.7%), gamification (7/312, 2.2%), tailoring (7/312, 2.2%), relaxation (4/312, 1.3%), breathing techniques (1/312, 0.3%), serious games (1/312, 0.3%), and others (7/312, 2.2%). On average, an app provided 3.34 (SD 2.29) functions (range 0-10). App description and developers’ or providers’ websites indicated that no app was certified according to the medical device law.

### Data Security and Privacy Features

Of the 312 assessed apps, 67 (21.5%) had an imprint/contact information, 60 (19.2%) provided a visible privacy policy, 25 (8.0%) were only accessible with a personalized log-in, 20 (6.4%) utilized a passive informed consent (eg, by continuing you accept our privacy policy), 15 (4.8%) contained an active informed consent (eg, active opt-in to data collection/transfer), 16 (4.8%) had a password option, and 5 (1.6%) gave information about the financial background or made conflicts of interest transparent. No app had embedded emergency features (eg, in case of an accident).

### App Quality

**Figure 3:**
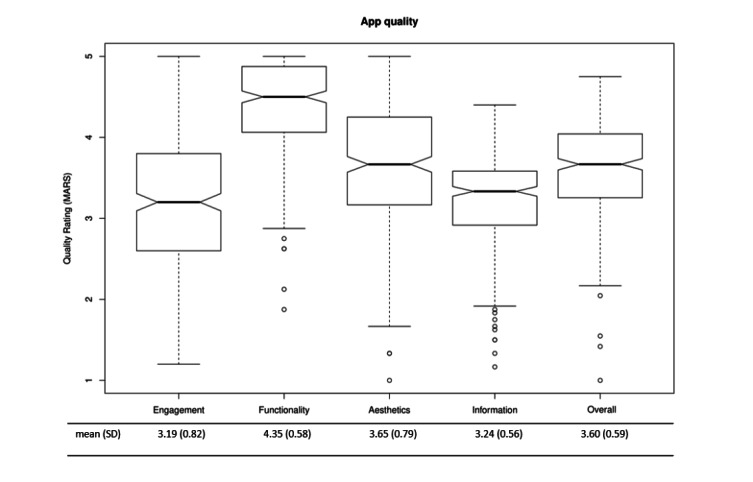
Quality of the apps. MARS: Mobile App Rating Scale.

The ICC agreement between the raters was high (ICC 0.87, 95% CI 0.86-0.874). The average overall quality of the apps for physical activity was 3.60 (SD 0.59) (range 1-4.75) with average quality of information (3.24 [SD 0.56], range 1.17-4.4) and engagement (3.19 [SD 0.82], range 1.2-5). Aesthetics (3.65 [SD 0.79], range 1-5) was good and functionality was excellent (4.35 [SD 0.58], range 1.88-5). Of all the 312 included apps, 10 (3.2%) reached a MARS score of above 4.5. The evidence item (based on app description, developer’s and provider’s websites) indicated that no app was scientifically evaluated. The MARS quality ratings are summarized in Figure 3. The overall subjective quality reached an average rating of 2.34 (SD 0.78) and the overall perceived impact on the user was rated as 2.32 (SD 0.60). The details can be found in Table 1. The 10 apps with the highest quality ratings are presented in Multimedia Appendix 1.

**Table 1:** Subjective quality ratings and ratings of the perceived impact on the users of mobile app rating scale.

Variable		Score, mean (SD)
**Subjective quality rating**		
	Recommendable^a^	2.67 (1.05)
	Use next 12 months^b^	2.38 (1.12)
	Pay^c^	1.27 (0.46)
	Star rating^d^	3.06 (0.89)
	Overall	2.34 (0.78)
**Perceived impact on user**		
	Increase awareness	2.16 (0.81)
	Increase knowledge	2.65 (0.90)
	Attitudes	2.26 (0.84)
	Fosters intention to change	2.47 (0.90)
	Empowers help-seeking	1.31 (0.62)
	Fosters behavior change	3.07 (0.88)
	Overall	2.32 (0.60)

^a^Rated on a 5-point scale (from 1: I would not recommend the app to anyone, to 5: I would recommend the app to everyone).

^b^Rated on a 5-point scale (1: never; 2: 1-2; 3: 3-10; 4: 10-50; 5: >50).

^c^Rated on a 3-point scale (1: I would not buy this app if it cost anything; 2: I might buy this app if it cost anything; 3: I would buy this app if it cost anything).

^d^Rated on a 5-point scale (from 1: one of the worst apps I have ever used, to 5: one of the best apps I have ever used).

### User and Expert Agreement Toward Quality

Small correlations were found between the user ratings in the stores and the MARS. The correlations are summarized in Table 2.

**Table 2:** Correlation between mobile app rating scale and user rating.

Mobile app rating scale dimension	User rating, *r* (95% CI)	*P* value^a^
Engagement	0.25 (0.11-0.38)	<.001
Functionality	0.15 (0.01-0.28)	.03
Aesthetics	0.14 (0.02-0.24)	.03
Information quality	0.14 (0.02-0.26)	.03
Overall	0.22 (0.08-0.35)	<.001

^a^Adjusted *P* value for multiple testing [35].

### Exploratory Regression Analysis

Exploratory regression analysis indicated that app quality could be predicted by the number of functions integrated into the app. Price and store had no predictive value. The results of the regression analyses are summarized in Table 3.

**Table 3:** Results of the regression analysis showing the predictors of app quality.

Predictor	β	SD	*t (df)*	*P* value	Adjusted *R*^*2*^ (%)
Price	.22	0.19	1.15 (310)	.25	0.00
Store	–.13	0.07	–1.94 (310)	.05	0.88
Number of functions/amount of content	.12	0.01	9.64 (310)	<.001	22.82

## Discussion

### Principal Findings

In this study, the quality, general characteristics, privacy and security features, and content/functions of apps that promote physical activity in the commercial European app stores were systematically assessed. The included 312 apps showed a moderate overall quality (3.60 [SD 0.59], range 1-4.75). Moreover, several apps showed very high ratings, and there was a large range of quality ratings. The assessments of the 10 best-rated apps are described in detail in Multimedia Appendix 1. Functionality was the dimension with the highest rating, followed by aesthetics, information quality, and engagement. These results corroborate those of Schoeppe and colleagues [22] who evaluated diet and physical activity apps for children and adolescents (overall quality, mean 3.6).

The apps offered a variety of different functions (15 out of 22 functions were used). On average, 3 functions were applied per app, and the most common ones were exercises, goal setting, and monitoring/tracking. This is partly in line with previous reviews for apps promoting physical activity [15,16,18] or weight management [29]. Functions differed from the most frequently used BCTs in another review that evaluated apps for diet, physical activity, and sedentary behavior developed for children and adolescents (the top 3 functions provided instructions, general encouragement, contingent [22]). This discrepancy might be explained by the different target groups. Studies have already shown that BCTs incorporated in apps for health behavior change differ between adults and children/adolescents [20]. The average number of the 3 applied functions was lower compared to that in other reviews that reported 5-8 comparable BCTs [15,16,19,22]. This could be due to the broader range of the included apps in this study.

No randomized controlled trial evaluating the effectiveness of one of the included apps could be identified. This lack of a solid evidence base for the use of health apps is in line with that reported in other systematic reviews of app quality (eg, older adults, mindfulness, depression, rheumatoid arthritis, and posttraumatic stress disorder) [36-40]. These systematic reviews showed that the proportion of the scientifically evaluated apps ranges between 0% and 4.8%. Overall, this indicates a gap between research and health practices. Although there are several randomized controlled trials that investigate the efficacy of sport app use to foster behavior change, these apps are not available in the app stores [41,42]. Of note, a vast majority of apps are downloaded from the Google Play store and App Store [43]. This might stem from the lack of sustainable structures at universities (eg, end of funding, frequent job changes). Furthermore, this imposes a risk for safe sport app use as the evidence base is the gold standard for assuring quality and efficacy. Moreover, data privacy and security features were also rated as low. Only 19.2% (60/312) of all the apps provided a privacy policy, and 21.5% (67/312) of the included apps provided any contact information or an imprint. All other privacy and security features were fulfilled by less than 20% (range 0-60) of all the apps. In contrast, Bondaronek and colleagues [19] stated that almost 70% of the 65 included physical activity apps had a privacy policy. This might be because they searched for the best-ranked apps. Taken together, the ratings of information quality (including correctness, credibility, and scientific evidence) and the ratings of data protection (including privacy policy, imprint, log-in, informed consent, password, conflicts of interest) reveal potential risks such as misinformation, adverse effects of app use, data misuse, or potential nonefficacy.

Average user ratings in the stores showed a significantly small correlation with the MARS ratings, which is in line with that reported in previous research [30,36]. However, several studies (including apps for weight management and chronic pain) could not identify an association [29,44]. This indicates that although user star ratings of physical activity apps might be used as an indicator for app quality, such an association should be evaluated for each indication separately. The results of our study suggest that mostly engagement might play a key role in the high user star rating that is contrary to previous results highlighting the impact of functionality [26,29]. Nevertheless, user star ratings should be interpreted with caution as end users lack the qualification to assess information quality. Furthermore, user star ratings lack credibility as they could originate from fictitious persons or they could refer to previous versions of the app [36].

The only relevant predictor for overall app quality was the number of functions, which is in line with previous results for apps reviews aiming at weight management, diet, physical activity, and sedentary behavior [16,22,29]. This association needs to be addressed in future studies, as it is highly likely that not only the number of functions but also their quality is crucial for overall quality. Furthermore, there might be an optimal number of functions; too many functions might be overwhelming, especially for inexperienced users. Owing to the lack of identified randomized controlled trials, no conclusions about the relationship between quality/functions and effectiveness or side effects can be drawn.

### Limitations and Future Research

In this review, apps were only searched in the Google Play store and App Store and, thus, this review does not cover all the available apps promoting physical activity. However, 90% of all the apps are downloaded in these stores [45]. Owing to the broad search strategy and no focus on only the most popular apps or a cut-off concerning user ratings, this review, including 312 apps, can be seen as comprehensive. The search terms were selected after a discussion between psychologists rather than sport scientists. However, EMM is a state-certified coach (Federal Sports Academy Austria). Furthermore, EMM and HB are experts in the field of app ratings with the MARS. The stated search terms provided a more comprehensive quality analysis of apps for physical activity than previous reviews (range 13-167) [15-18]. Since new apps are being developed rapidly and the content of existing apps might have changed, the presented results can only be seen as a snapshot of the current state of the offered apps. Meanwhile, several new apps may be available and some of the included apps may be unavailable by now or may have been updated. Furthermore, apps were not tested for several hours or days. Thus, some features may not have been discovered, and some obstacles may have reminded hidden. Previous reviews evaluating apps for physical activity [15,16,29] assessed BCTs that are common to many health behavior theories [21]. Even though some of these BCTs were included in the content and functions of this review (eg, self-monitoring, feedback on performance, goal setting, or provision of information), a comparison to results concerning BCTs of previous studies is limited. The comparability between systematic app reviews in different health domains should be enhanced by using the functions included in the MARS [38,39,44]. In this systematic review, privacy and security measures were assessed on a descriptive level. Data security and privacy information were only checked based on information within the app. An in-depth analysis of privacy and security features and more elaborated strategies (eg, evaluating whether data collection and transfer are conducted according to privacy policies) are needed [28]. Future studies should extend the findings of this study by using such procedures. Lastly, it should be highlighted that the regression analyses in this study were exploratory. Thus, the results should be interpreted carefully. Confirmatory studies with adequate study designs and power are needed to identify features that are crucial elements for high app quality. Looking at the number of functions—as specified in the exploratory analyses in this study—or investigating persuasive design might be promising to begin with [46,47].

### Conclusions

There is a wide range of apps offered to foster physical activity and they show overall moderate quality. High-quality apps have been presented in Multimedia Appendix 1. However, users should be aware of the broad quality range, the lack of evidence, and low ratings in privacy and security features. Thus, recommendations for the use of physical activity apps can only be given with major limitations. The contents and functions correlated positively with quality ratings. Furthermore, user ratings showed small correlations to the quality ratings and might be a limited indicator for end users. However, it seems necessary that developers use evidence-based content and scientifically developed and evaluated apps find their way into the app stores. Since the field of mobile health is rapidly growing, there is a need for continuous up-to-date evaluations of apps to provide and inform end users about data protection, privacy regulations, and evidence base. Central databases such as digital apothecaries [48-50] could help the user find high-quality apps and be protected against misinformation and abuse. However, there is also a need for novel methodological frameworks such as continuous evaluations [51] that allow for the assessment of multiple or evolving app versions.

## Appendix

Multimedia Appendix 1 Top 10 apps with the highest quality ratings.

## Abbreviations

BCT: behavior change technique
ICC: intraclass correlation coefficient
MARS-G: German version of the Mobile App Rating Scale

## References

[1] The world health report 2002: Reducing risks, promoting healthy life. World Health Organization.

[2] Lee I-M, Shiroma EJ, Lobelo F, Puska P, Blair SN, Katzmarzyk PT (2012). Effect of physical inactivity on major non-communicable diseases worldwide: an analysis of burden of disease and life expectancy. The Lancet.

[3] Global recommendations on physical activity for health. World Health Organization.

[4] Hallal PC, Andersen LB, Bull FC, Guthold R, Haskell W, Ekelund U (2012). Global physical activity levels: surveillance progress, pitfalls, and prospects. The Lancet.

[5] Health and development through physical activity and sport. World Health Organization.

[6] Galper DI, Trivedi MH, Barlow CE, Dunn AL, Kampert JB (2006). Inverse association between physical inactivity and mental health in men and women. Med Sci Sports Exerc.

[7] Physical activity guidelines advisory committee scientific report 2018. U.S. Department of Health and Human Services.

[8] Kuhn B, Amelung V, Albrecht U.-V. (2016). Kapitel 4. Gesundheits-Apps und besondere Herausforderungen. Chancen und Risiken von Gesundheits-Apps (CHARISMHA).

[9] Lindhiem O, Bennett CB, Rosen D, Silk J (2015). Mobile technology boosts the effectiveness of psychotherapy and behavioral interventions: A meta-analysis. Behavior Modification.

[10] Krebs P, Duncan DT (2015). Health app use among US mobile phone owners: A national survey. JMIR mHealth and uHealth.

[11] Carroll JK, Moorhead A, Bond R, LeBlanc WG, Petrella RJ, Fiscella K (2017). Who uses mobile phone health apps and does use matter? A secondary data analytics approach. Journal of Medical Internet Research.

[12] Coughlin SS, Whitehead M, Sheats JQ, Mastromonico J, Smith S (2016). A review of smartphone applications for promoting physical activity. Jacobs Journal of Community Medicine.

[13] Romeo A, Edney S, Plotnikoff R, Curtis R, Ryan J, Sanders I, Crozier A, Maher C (2019). Can smartphone apps increase physical activity? Systematic review and meta-analysis. Journal of Medical Internet Research.

[14] Mateo GF, Granado-Font E, Ferré-Grau C, Montaña-Carreras X (2015). Mobile phone apps to promote weight loss and increase physical activity: A systematic review and meta-analysis. Journal of Medical Internet Research.

[15] Middelweerd A, Mollee JS, van der Wal CN, Brug J, te Velde SJ (2014). Apps to promote physical activity among adults: A review and content analysis. International Journal of Behavioral Nutrition and Physical Activity.

[16] Direito A, Dale LP, Shields E, Dobson R, Whittaker R, Maddison R (2014). Do physical activity and dietary smartphone applications incorporate evidence-based behaviour change techniques?. BMC Public Health.

[17] Conroy DE, Yang C-H, Maher JP (2014). Behavior change techniques in top-ranked mobile apps for physical activity. American Journal of Preventive Medicine.

[18] Lyons EJ, Lewis ZH, Mayrsohn BG, Rowland JL (2014). Behavior change techniques implemented in electronic lifestyle activity monitors: A systematic content analysis. Journal of Medical Internet Research.

[19] Bondaronek P, Alkhaldi G, Slee A, Hamilton FL, Murray E (2018). Quality of publicly available physical activity apps: Review and content analysis. JMIR mHealth and uHealth.

[20] Brannon EE, Cushing CC (2015). A systematic review: Is there an app for that? Translational science of pediatric behavior change for physical activity and dietary interventions. Journal of Pediatric Psychology.

[21] Abraham C, Michie S (2008). A taxonomy of behavior change techniques used in interventions. Health Psychology.

[22] Schoeppe S, Alley S, Rebar AL, Hayman M, Bray NA, van Lippevelde W, Gnam J-P, Bachert P, Direito A, Vandelanotte C (2017). Apps to improve diet, physical activity and sedentary behaviour in children and adolescents: A review of quality, features and behaviour change techniques. International Journal of Behavioral Nutrition and Physical Activity.

[23] Stoyanov SR, Hides L, Kavanagh DJ, Zelenko O, Tjondronegoro D, Mani M (2015). Mobile app rating scale: A new tool for assessing the quality of health mobile apps. JMIR mHealth and uHealth.

[24] McKay FH, Cheng C, Wright A, Shill J, Stephens H, Uccellini M (2018). Evaluating mobile phone applications for health behaviour change: A systematic review. Journal of Telemedicine and Telecare.

[25] Albrecht U-V (2016). Chancen und Risiken von Gesundheits-Apps (CHARISMHA).

[26] Armstrong S (2017). Which app should I use? Patients and doctors are making increasing use of health apps, but there is little guidance about how well they work. British Journal of Sports Medicine.

[27] Grundy Q, Chiu K, Bero L (2019). Commercialization of user data by developers of medicines-related apps: A content analysis. Journal of General Internal Medicine.

[28] Huckvale K, Torous J, Larsen ME (2019). Assessment of the data sharing and privacy practices of smartphone apps for depression and smoking cessation. JAMA Network Open.

[29] Bardus M, van Beurden SB, Smith JR, Abraham C (2016). A review and content analysis of engagement, functionality, aesthetics, information quality, and change techniques in the most popular commercial apps for weight management. International Journal of Behavioral Nutrition and Physical Activity.

[30] Terhorst Y, Philippi P, Sander LB, Schultchen D, Paganini S, Bardus M, Santo K, Knitza J, Machado GC, Schoeppe S, Bauereiß N, Portenhauser A, Domhardt M, Walter B, Krusche M, Baumeister H, Messner E-M (2020). Validation of the Mobile Application Rating Scale (MARS). PLoS One.

[31] Messner E-M, Terhorst Y, Barke A, Baumeister H, Stoyanov S, Hides L, Kavanagh D, Pryss R, Sander L, Probst T (2020). The German version of the Mobile App Rating Scale (MARS-G): Development and validation study. JMIR mHealth and uHealth.

[32] Features of the rating scale. Mobile App Rating Scale-German version.

[33] Koo TK, Li MY (2016). A guideline of selecting and reporting intraclass correlation coefficients for reliability research. Journal of Chiropractic Medicine.

[34] Portney LG, Watkins MP (2009). Foundations of Clinical Research: Applications to Practice.

[35] Holm S (1979). A simple sequentially rejective multiple test procedure. Scandinavian Journal of Statistics.

[36] Portenhauser AA, Terhorst Y, Schultchen D, Sander LB, Denkinger MD, Stach M, Waldherr N, Dallmeier D, Baumeister H, Messner E-M (2021). Mobile Apps for Older Adults: Systematic Search and Evaluation Within Online Stores. JMIR Aging.

[37] Schultchen D, Terhorst Y, Holderied T, Stach M, Messner E-M, Baumeister H, Sander LB (2020). Stay present with your phone: A systematic review and standardized rating of mindfulness apps in european app stores. International Journal of Behavioral Medicine.

[38] Sander LB, Schorndanner J, Terhorst Y, Spanhel K, Pryss R, Baumeister H, Messner E-M (2020). 'Help for trauma from the app stores?' A systematic review and standardised rating of apps for Post-Traumatic Stress Disorder (PTSD). Eur J Psychotraumatol.

[39] Terhorst Y, Rathner E-M, Baumeister H, Sander LB (2018). «Hilfe aus dem App-Store?»: Eine systematische Übersichtsarbeit und Evaluation von Apps zur Anwendung bei Depressionen. Verhaltenstherapie.

[40] Knitza J, Tascilar K, Messner E-M, Meyer M, Vossen D, Pulla A, Bosch P, Kittler J, Kleyer A, Sewerin P, Mucke J, Haase I, Simon D, Krusche M (2019). German mobile apps in rheumatology: Review and analysis using the Mobile Application Rating Scale (MARS). JMIR mHealth and uHealth.

[41] van Reijen M, Vriend I, van Mechelen W, Verhagen EA (2018). Preventing recurrent ankle sprains: Is the use of an app more cost-effective than a printed booklet? Results of a RCT. Scandinavian Journal Medicine & Science in Sports.

[42] Edney SM, Olds TS, Ryan JC, Vandelanotte C, Plotnikoff RC, Curtis RG, Maher CA (2020). A social networking and gamified app to increase physical activity: Cluster RCT. American Journal of Preventive Medicine.

[43] Number of apps available in leading app stores as of 4th quarter 2020. Statista.

[44] Terhorst Y, Messner E-M, Schultchen D, Paganini S, Portenhauser A, Eder A-E, Bauer M, Papenhoff M, Baumeister H, Sander LB (2021). Systematic evaluation of content and quality of English and German pain apps in European app stores. Internet Interv.

[45] Mobile operating system market share worldwide 1999-2020. StatCounter.

[46] Baumeister H, Kraft R, Baumel A, Pryss R, Messner E-M, Baumeister H, Montag C (2019). Persuasive e-health design for behavior change. Digital Phenotyping and Mobile Sensing: New developments in Psychoinformatics.

[47] Baumel A, Yom-Tov E (2018). Predicting user adherence to behavioral eHealth interventions in the real world: Examining which aspects of intervention design matter most. Translational Behavioral Medicine.

[48] Muñoz RF, Chavira DA, Himle JA, Koerner K, Muroff J, Reynolds J, Rose RD, Ruzek JI, Teachman BA, Schueller SM (2018). Digital apothecaries: A vision for making health care interventions accessible worldwide. Mhealth.

[49] Apps and digital health resources reviewed by experts. One Mind PsyberGuide.

[50] kvappradar: Doctors and psychotherapists rate health apps. Central Institute for Statutory Health Insurance in the Federal Republic of Germany.

[51] Mohr DC, Burns MN, Schueller SM, Clarke G, Klinkman M (2013). Behavioral intervention technologies: Evidence review and recommendations for future research in mental health. General Hospital Psychiatry.

