# Supplementary material for: Quality of Physical Activity Apps: Systematic Search in App Stores and Content Analysis
Source: JMIR Mhealth Uhealth. 2021 Jun 9;9(6):e22587. doi: 10.2196/22587 (PMC8262667; doi:10.2196/22587)
Supplement: Multimedia Appendix 1 [file mhealth_v9i6e22587_app1.docx]

**Online Supplemental Material 1**

Content A

*Highest ratings for app quality according to the MARS*

| **Name** | **Engagement** | **Functionality** | **Aesthetics** | **Information** | **Overall** |
| --- | --- | --- | --- | --- | --- |
| iMuscle 2 | 5.00 | 5.00 | 5.00 | 4.00 | 4.75 |
| Sweat: Kayla Itsines Fitness | 4.80 | 4.88 | 5.00 | 4.08 | 4.69 |
| Stoppani Shortcut to Strength | 4.50 | 5.00 | 5.00 | 4.25 | 4.69 |
| Fitbit Coach | 5.00 | 4.88 | 5.00 | 3.58 | 4.61 |
| 8fit Fitness- & Ernährungsplan | 4.80 | 4.63 | 5.00 | 4.00 | 4.61 |
| FitWell Personal Fitness Coach | 4.90 | 4.75 | 5.00 | 3.75 | 4.60 |
| Endomondo Sports Tracker | 4.50 | 5.00 | 4.67 | 4.08 | 4.56 |
| 8fit Workouts & Meal Planner | 4.50 | 4.88 | 5.00 | 3.83 | 4.55 |
| Sworkit: Workouts & Fitness Plans | 4.30 | 4.75 | 5.00 | 4.08 | 4.53 |
| Yogaia: Live Yoga Workouts | 4.50 | 4.88 | 5.00 | 3.75 | 4.53 |

Content B

*Ratings for app engagement according to the MARS*

| **Name** | **Fun** | **Interest** | **Individual Adaptability** | **Interactivity** | **Target Group** | **Engagement Overall** |
| --- | --- | --- | --- | --- | --- | --- |
| iMuscle 2 | 5.00 | 5.00 | 5.00 | 5.00 | 5.00 | 5.00 |
| Sweat: Kayla Itsines Fitness | 5.00 | 5.00 | 4.50 | 4.50 | 5.00 | 4.80 |
| Stoppani Shortcut to Strength | 5.00 | 5.00 | 4.00 | 3.50 | 5.00 | 4.50 |
| Fitbit Coach | 5.00 | 5.00 | 5.00 | 5.00 | 5.00 | 5.00 |
| 8fit Fitness- & Ernährungsplan | 5.00 | 5.00 | 5.00 | 4.50 | 4.50 | 4.80 |
| FitWell Personal Fitness Coach | 4.50 | 5.00 | 5.00 | 5.00 | 5.00 | 4.90 |
| Endomondo Sports Tracker | 5.00 | 5.00 | 4.00 | 3.50 | 5.00 | 4.50 |
| 8fit Workouts & Meal Planner | 4.50 | 4.50 | 4.50 | 4.00 | 5.00 | 4.50 |
| Sworkit: Workouts & Fitness Plans | 4.50 | 4.50 | 4.00 | 3.50 | 5.00 | 4.30 |
| Yogaia: Live Yoga Workouts | 4.50 | 4.50 | 4.00 | 4.50 | 5.00 | 4.50 |

Content C

*Ratings for app functionality according to the MARS*

| **Name** | **Performance** | **Usability** | **Navigation** | **Gestural Design** | **Functionality Overall** |
| --- | --- | --- | --- | --- | --- |
| iMuscle 2 | 5.00 | 5.00 | 5.00 | 5.00 | 5.00 |
| Sweat: Kayla Itsines Fitness | 5.00 | 5.00 | 5.00 | 4.50 | 4.88 |
| Stoppani Shortcut to Strength | 5.00 | 5.00 | 5.00 | 5.00 | 5.00 |
| Fitbit Coach | 4.50 | 5.00 | 5.00 | 5.00 | 4.88 |
| 8fit Fitness- & Ernährungsplan | 4.00 | 5.00 | 5.00 | 4.50 | 4.63 |
| FitWell Personal Fitness Coach | 4.50 | 4.50 | 5.00 | 5.00 | 4.75 |
| Endomondo Sports Tracker | 5.00 | 5.00 | 5.00 | 5.00 | 5.00 |
| 8fit Workouts & Meal Planner | 5.00 | 5.00 | 5.00 | 4.50 | 4.88 |
| Sworkit: Workouts & Fitness Plans | 4.50 | 5.00 | 5.00 | 4.50 | 4.75 |
| Yogaia: Live Yoga Workouts | 5.00 | 5.00 | 5.00 | 4.50 | 4.88 |

Content D

*Ratings for app aesthetics according to the MARS*

| **Name** | **Layout** | **Graphics** | **Visual Appeal** | **Aesthetics Overall** |
| --- | --- | --- | --- | --- |
| iMuscle 2 | 5.00 | 5.00 | 5.00 | 5.00 |
| Sweat: Kayla Itsines Fitness | 5.00 | 5.00 | 5.00 | 5.00 |
| Stoppani Shortcut to Strength | 5.00 | 5.00 | 5.00 | 5.00 |
| Fitbit Coach | 5.00 | 5.00 | 5.00 | 5.00 |
| 8fit Fitness- & Ernährungsplan | 5.00 | 5.00 | 5.00 | 5.00 |
| FitWell Personal Fitness Coach | 5.00 | 5.00 | 5.00 | 5.00 |
| Endomondo Sports Tracker | 5.00 | 4.50 | 4.50 | 4.67 |
| 8fit Workouts & Meal Planner | 5.00 | 5.00 | 5.00 | 5.00 |
| Sworkit: Workouts & Fitness Plans | 5.00 | 5.00 | 5.00 | 5.00 |
| Yogaia: Live Yoga Workouts | 5.00 | 5.00 | 5.00 | 5.00 |

Content E

*Ratings for app information according to the MARS*

| **Name** | **Accuracy Description** | **Goals** | **Quality of Infor-mation** | **Quantity of Infor-mation** | **Quality of Visual Infor-mation** | **Credi-bility** | **Infor-mation Overall** |
| --- | --- | --- | --- | --- | --- | --- | --- |
| iMuscle 2 | 4.50 | 5.00 | 4.50 | 4.50 | 4.50 | 1.00 | 4.00 |
| Sweat: Kayla Itsines Fitness | 4.50 | 4.50 | 5.00 | 4.50 | 5.00 | 1.00 | 4.08 |
| Stoppani Shortcut to Strength | 5.00 | 5.00 | 5.00 | 4.50 | 5.00 | 1.00 | 4.25 |
| Fitbit Coach | 4.00 | 4.00 | 4.00 | 4.00 | 4.50 | 1.00 | 3.58 |
| 8fit Fitness- & Ernährungsplan | 5.00 | 4.00 | 4.50 | 4.50 | 5.00 | 1.00 | 4.00 |
| FitWell Personal Fitness Coach | 4.00 | 4.50 | 4.50 | 4.00 | 4.50 | 1.00 | 3.75 |
| Endomondo Sports Tracker | 5.00 | 5.00 | 4.50 | 4.00 | 5.00 | 1.00 | 4.08 |
| 8fit Workouts & Meal Planner | 4.50 | 4.50 | 4.50 | 4.00 | 4.50 | 1.00 | 3.83 |
| Sworkit: Workouts & Fitness Plans | 5.00 | 5.00 | 4.50 | 4.00 | 5.00 | 1.00 | 4.08 |
| Yogaia: Live Yoga Workouts | 4.00 | 4.00 | 4.50 | 4.00 | 5.00 | 1.00 | 3.75 |
